# Longitudinal assessment of cognitive decline in hemodialysis patients: unveiling sex disparities

**DOI:** 10.1093/ckj/sfag340

**Published:** 2026-10-07

**Authors:** Hannah Schäfer, Matthias Braunisch, Roman Günthner, Claudius Küchle, Michael Basta-Wunderle, Bernhard Haller, Uwe Heemann, Christoph Schmaderer, Susanne Angermann

**Affiliations:** School of Medicine and Health, Department of Nephrology, TUM University Hospital, Technical University of Munich, Munich, Germany; School of Medicine and Health, Department of Nephrology, TUM University Hospital, Technical University of Munich, Munich, Germany; School of Medicine and Health, Department of Nephrology, TUM University Hospital, Technical University of Munich, Munich, Germany; School of Medicine and Health, Department of Nephrology, TUM University Hospital, Technical University of Munich, Munich, Germany; School of Medicine and Health, Department of Nephrology, TUM University Hospital, Technical University of Munich, Munich, Germany; School of Medicine and Health, Institute of AI and Informatics in Medicine, TUM University Hospital, Technical University of Munich, Munich, Germany; School of Medicine and Health, Department of Nephrology, TUM University Hospital, Technical University of Munich, Munich, Germany; School of Medicine and Health, Department of Nephrology, TUM University Hospital, Technical University of Munich, Munich, Germany; School of Medicine and Health, Department of Nephrology, TUM University Hospital, Technical University of Munich, Munich, Germany; Internistische Gemeinschaftspraxis und Dialysezentrum Landshut, Landshut, Germany

**Keywords:** arterial stiffness, chronic hemodialysis, cognitive decline, gender, vascular calcification

## Abstract

**Background:**

Numerous studies have delved into the prevalence, risk factors, and outcomes associated with cognitive impairment in hemodialysis patients. However, a scarcity of data exists regarding the trajectory of cognitive decline and potential contributing factors.

**Methods:**

We recruited 247 prevalent hemodialysis patients for this study. Baseline and follow-up cognitive assessments were conducted in 90 patients using the Montreal Cognitive Assessment (MoCA) at a 2-year interval. To identify potential predictors of cognitive decline, we analyzed traditional risk factors alongside dialysis-related variables. Additionally, surrogate parameters of vascular calcification and arterial stiffness, such as T50 values and pulse wave velocity, were investigated.

**Results:**

After a 2-year follow-up period (24.3 ± 1.05 months), MoCA test results revealed a significant decline in cognitive function (−1.8 ± 3.2 points, *P* < 0.001). Overall, 37 patients (41%) exhibited cognitive decline, defined as a decrease in MoCA score of >2 points, indicating disease progression. The median change in MoCA score differed significantly between males (−3.0, range 0.0 to −5 points) and females (0.0, range 1.0 to −1.75 points, *P* = 0.002). This finding was corroborated by multivariable analyses adjusted for age, educational level, diabetes and blood pressure measurements (regression coefficient −2.5, *P* = 0.006). The association was further confirmed in a comparison between patients with stable and declining cognitive function (regression coefficient 2.65, odds ratio 14.2, *P* = 0.007). None of the other variables reached statistical significance.

**Conclusions:**

Our findings underscore a substantial deterioration in cognitive function over time among hemodialysis patients. Notably, our data reveal a more rapid decline in cognitive function among males, shedding light on previously unrecognized sex differences in this patient population.

## Graphical Abstract

**Graphical Abstract:**
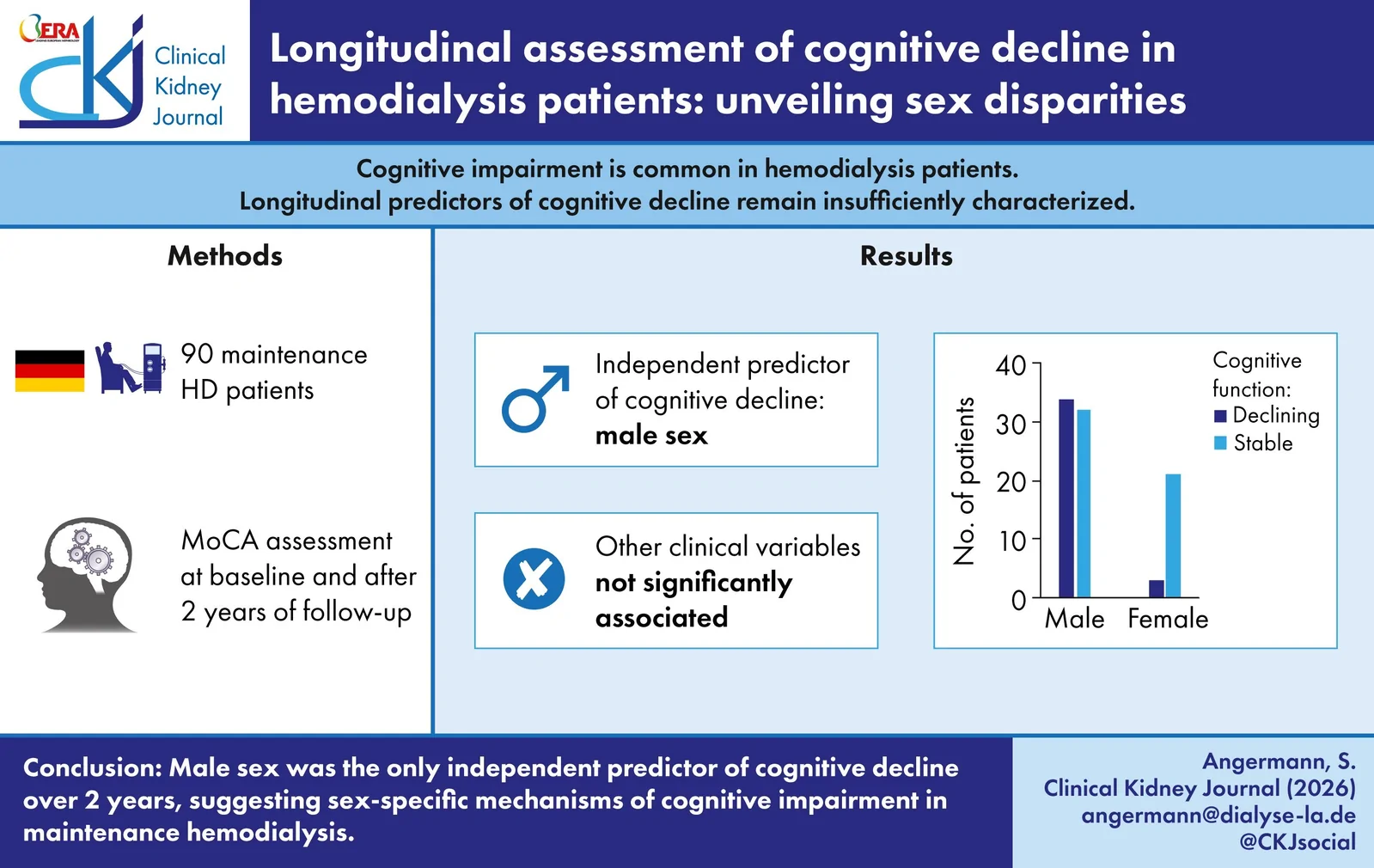

KEY LEARNING POINTS **What was known:**

- Cognitive impairment has become a well-recognized problem among hemodialysis patients contributing to even worse prognosis. Longitudinal data on the course of cognitive decline and associated risk factors are sparse.

**This study adds:**

- In the current study, there was a significant deterioration of cognitive function in 90 hemodialysis patients after a 2-year interval based on repeated Montreal Cognitive Assessment (MoCA) testing. This decline was more pronounced in males, identifying male sex as the only risk factor for progressive disease in adjusted analyses.

**Potential impact:**

- The observed relationship points to previously unrecognized sex differences in the progression of cognitive impairment among hemodialysis patients. Further research is warranted to determine whether male sex is an independent risk factor for disease progression and to clarify the mechanisms underlying this association.

## INTRODUCTION

The prevalence and adverse outcomes associated with cognitive impairment are widely acknowledged in the end-stage renal disease (ESRD) patient population. However, uncertainties persist regarding the pathogenesis and progression of the disease. Growing evidence suggests a vascular origin of cognitive impairment and dementia in hemodialysis patients [1].

Several longitudinal studies on cognitive function in hemodialysis cohorts have indicated a significant decline over time [2–5]. However, some studies have reported stable or improved cognitive function [6–8]. Identified contributors to progression in these studies include older age and frailty. In addition, reductions in mean cerebral blood flow during hemodialysis sessions have been linked to worsening cognitive function [5, 8]. Overall, the available evidence indicates that the trajectory of cognitive function in hemodialysis patients remains incompletely characterized, and the factors associated with cognitive decline are not yet fully understood.

Therefore, the primary objective of our study is to investigate the trajectory of cognitive function in hemodialysis patients and identify risk factors for its deterioration.

## MATERIALS AND METHODS

### Patient selection

In this prospective, longitudinal, observational cohort study, we enrolled dialysis patients from six different dialysis centers in and around Munich between June 2011 and July 2014. This study is part of the rISk strAtification in end-stage renal disease (ISAR) study, registered at ClinicalTrials.gov (identifier number: NCT01152892). The trial aimed to characterize the cardiovascular phenotype of the study subjects and identify associations between novel and established risk factors and cardiovascular disease. The detailed study protocol was published in BMC Nephrology [9]. Within this study, cognitive function was assessed repeatedly. Inclusion criteria were age >18 years and chronic hemodialysis for >3 months. Patients with systemic infections, peritoneal dialysis, electrolyte imbalances, pregnancy, or malignant disease with a survival prognosis of <2 years were excluded. Additionally, patients were excluded if German was not their mother tongue, if they had a motor disorder of the dominant hand, aphasia, or amaurosis, as these conditions could interfere with the standardized administration and validity of cognitive testing. Eligible patients underwent initial and follow-up Montreal Cognitive Assessment (MoCA) testing and, upon providing written informed consent, were included in the analysis. The corresponding ethics committee approved the study (No. 344/15 s). All clinical investigations adhered to the principles of the Declaration of Helsinki, sixth revision.

### Cognitive testing

Cognitive function was evaluated using the MoCA, a brief screening tool. The MoCA was selected over the Mini Mental State Examination (MMSE) because it assesses executive functions, which are frequently impaired early in hemodialysis patients with cognitive dysfunction. In addition, the MoCA has been validated against detailed neuropsychological test batteries and has shown robust diagnostic performance [10]. With a maximum score of 30 points, results below 26 points are considered pathological in the general population. Previous studies have suggested a more sensitive cut-off value of 24 points for hemodialysis patients [10, 11], and, accordingly, MoCA test results below 24 points were classified as pathological in this study. To assess risk factors for cognitive decline over time, we utilized the absolute difference in MoCA test results between baseline and follow-up, but also a cut-off value of 2 points (chosen by the authors) classifying patients into two groups: patients with a decrease of >2 points were categorized as having declining cognitive function (referred to as the ‘dCF group’), while others were considered stable (the ‘sCF group’). Given the unclear optimal timing for neuropsychological testing in hemodialysis patients [12–15], cognitive assessments were conducted under standardized conditions (before dialysis and alone in a separate room) on a midweek dialysis day, as previously published [16].

### Risk factors

To evaluate factors associated with cognitive function decline, data on various risk factors were collected, including traditional risk factors at baseline (age, sex, educational level, and cardiovascular risk factors), dialysis-associated risk factors (dialysis vintage, time on dialysis, ultrafiltration, and Kt/V as an equivalent for dialysis efficacy), and risk factors for vascular calcification (calcium, phosphate, parathyroid hormone, and T50 values). Information on nicotine use was limited to current smoking status. Hemodynamic factors, including heart rate, blood pressure (systolic, diastolic, and mean arterial pressure), and pulse wave velocity (PWV), were also considered. Laboratory values were extracted from the most recent quarterly examination at the corresponding dialysis center at baseline. The calcification propensity of serum was assessed using the T50-Test at baseline. Serum was collected prior to a midweek dialysis session at time of enrollment and processed as previously described by Pasch *et al*. and Lorenz *et al*. [17, 18]. The measurement of heart rate, blood pressure, and PWV were conducted as published previously [19]. PWV was measured noninvasively as a continuous measurement over 24 h beginning before dialysis using the Mobil-O-Graph ® by IEM Healthcare. Calculations of PWV were derived from an oscillometric pulse contour analysis from the upper arm where the pressure cuff was attached. The single values were integrated to a 24 h PWV.

### Statistical analysis

Statistical analysis was conducted using IBM SPSS Statistics 27–29 software. MoCA values not adjusted for educational level were used for all calculations [20]. Educational level was included as a separate variable in our analyses based on the MoCA-recommended cut-off of 12 years of education, allowing for a rough approximation of the different levels within the German educational system [11, 19, 21]. Descriptive statistics were used to calculate demographic and clinical characteristics with mean ± standard deviations (SD) stated for normally distributed data (based on Shapiro–Wilk normality test). Paired *t*-tests compared differences between MoCA total scores and subcategories at baseline and the 2-year follow-up. Based on the change in MoCA total score (>2 points), patients were grouped into dCF and sCF groups, as described above. Correlations between absolute MoCA difference and risk factors were tested using Pearson’s and Spearman-rank correlation. Group differences were analyzed by unpaired *t*-test, Mann–Whitney-*U*, and *χ*^2^-test, as appropriate. Regression models (linear or logistic, as appropriate), with covariates age, peripheral systolic and diastolic blood pressure, mean blood pressure, diabetes, and educational level, were fitted to the data with either the MoCA difference or dCF as dependent variables. To account for potential selection bias between patients with and without follow-up examination, group comparisons were performed using unpaired *t*-tests and *χ*² tests, as appropriate. Sensitivity analyses were conducted to evaluate the potential influence of survivor bias by imputing alternative cognitive outcomes for participants who died before follow-up, including a MoCA score of 0, 50% of the baseline MoCA score, and last observation carried forward (LOCF).

## RESULTS

### Patients

A total of 247 patients underwent cognitive testing using the MoCA at baseline, and 90 patients participated in the follow-up assessment. The reasons for drop-out were as follows: 37 patients died, 11 received kidney transplants, and 6 were lost to follow-up. An additional 103 did not consent to repeated testing (Fig. 1). Patients without follow-up examination showed a trend toward lower baseline MoCA scores compared with those with follow-up (23.4 ± 4.1 vs. 24.3 ± 3.8 points, *P* = 0.056). The only statistically significant difference was observed for hypercholesterolemia (*P* = 0.043), which was more prevalent in patients without follow-up (71% vs. 58%; Table S1). Sensitivity analyses addressing mortality-related missing data demonstrated that the estimated magnitude of cognitive decline depended on the assumptions made for deceased participants. While the overall conclusions remained unchanged, imputing poor cognitive outcomes resulted in substantially greater declines than those observed in the complete-case analysis (Table S2).

**Figure 1:**
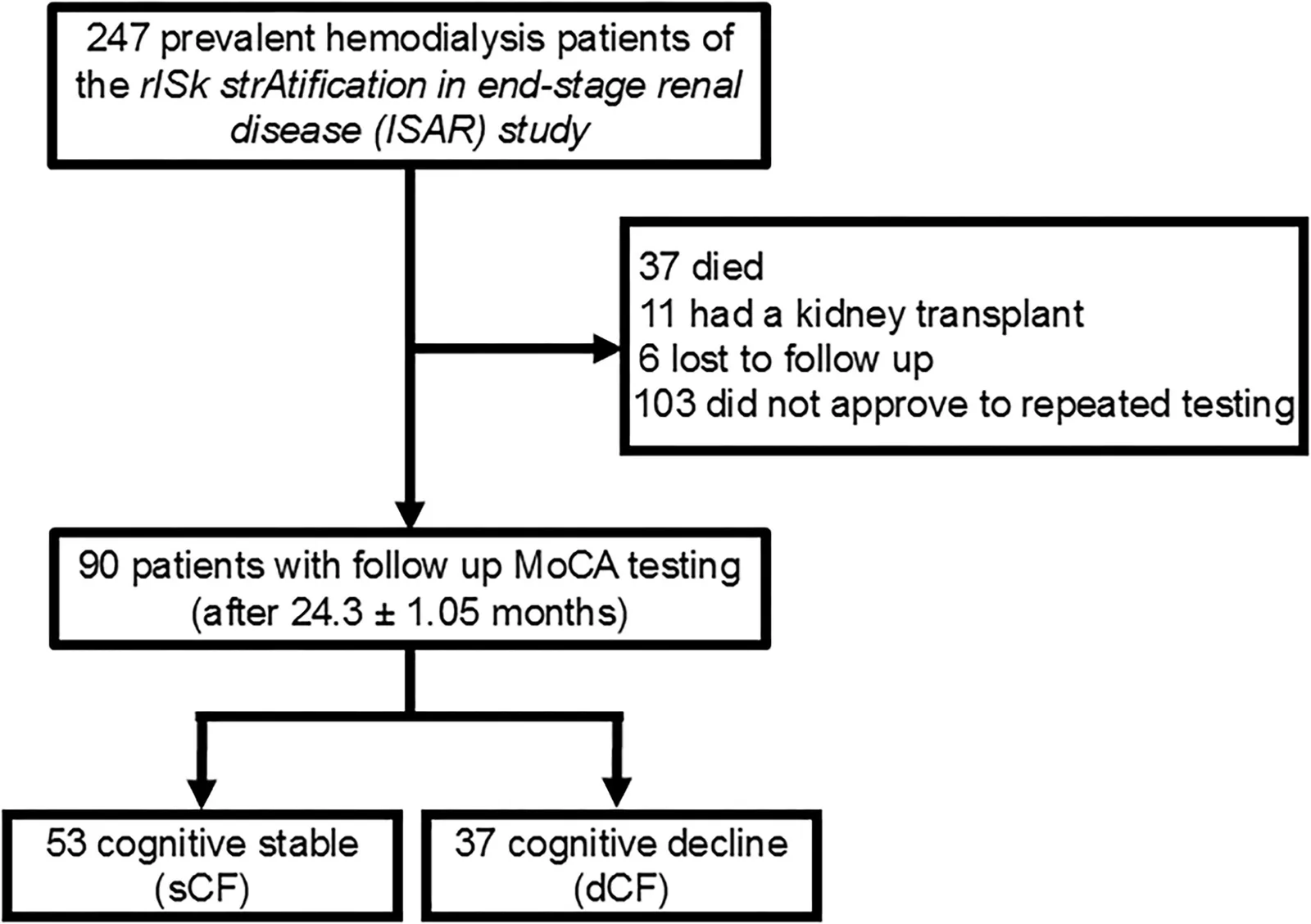
Flow chart showing patient attrition from baseline to follow-up MoCA assessment. Among the 90 patients with available follow-up data, cognitive trajectories were classified as declining cognitive function (dCF) or stable cognitive function (sCF), based on a decrease of more than 2 points in the total MoCA score.

At enrollment, the mean age was 62.6 years, with 72.5% of study participants being males, and 45% having >12 years of formal education. Most patients presented with at least one cardiovascular risk factor, primarily hypertension. The mean dialysis vintage was 82 months. Baseline demographic and clinical characteristics, as well as dialysis-associated risk factors, risk factors for vascular calcification, and hemodynamics, are summarized in Table 1.

**Table 1:** Demographic and clinical characteristics and factors associated with cognitive decline.

Traditional risk factors	
Age (years), mean ± SD	62.6 ± 14.8
Sex m/f, *n* (%)	66 (73)/24 (27)
Educational level <12 years, *n* (%)	50 (55)
Arterial hypertension, *n* (%)	81 (89)
Diabetes mellitus, *n* (%)	26 (29)
Hypercholesterolemia, *n* (%)	47 (52)
Current smokers, *n* (%)	16 (18)
**Dialysis associated risk factors**	
Dialysis vintage (months), median (range)	41 (21–78)
Time on dialysis (h), mean ± SD	4.5 ± 0.9
Ultrafiltration volume (l), mean ± SD	1.8 ± 1.0
Kt/V, mean ± SD	1.6 ± 0.5
**Risk factors for vascular calcification**	
Calcium (mmol/l), mean ± SD	2.3 ± 0.2
Phosphate (mmol/l), mean ± SD	5.3 ± 1.3
PTH (pg/ml), median (range)	244 (59−389)
T_50_ (min), mean ± SD	244 ± 76
**Hemodynamics**	
HR (bpm), mean ± SD	73 ± 11
pSBP (mmHg), mean ± SD	111 ± 13
pMAP (mmHg), mean ± SD	88 ± 11
pDBP (mmHg), mean ± SD	76 ± 11
PWV (m/s), mean ± SD	9.0 ± 2.2

bpm, beats per minute; HR, heart rate; m/f, male/female; pDBP, 24 h peripheral diastolic blood pressure; pMAP, 24 h peripheral mean arterial pressure; pSBP, 24 h peripheral systolic blood pressure; T50, T50-test

### Evaluation of cognitive function

MoCA total scores were significantly lower during the follow-up testing conducted 24.3 ± 1.05 months after the baseline examination (mean MoCA total score of 24.8 ± 3.7 points at baseline and 23.0 ± 4.9 points at follow-up, *P* < 0.001). The change overtime in MoCA total scores is depicted in Fig. 2. The most notable deterioration occurred in the subcategories of visuospatial/executive function, attention, abstraction, and orientation (Table 2, *P* < 0.001, *P* = 0.008, *P* < 0.001, and *P* < 0.001, respectively). Memory function/recall, language/verbal fluency and naming showed no significant difference between both time points (*P* = 0.14, *P* = 0.16 and *P* = 0.59).

**Figure 2:**
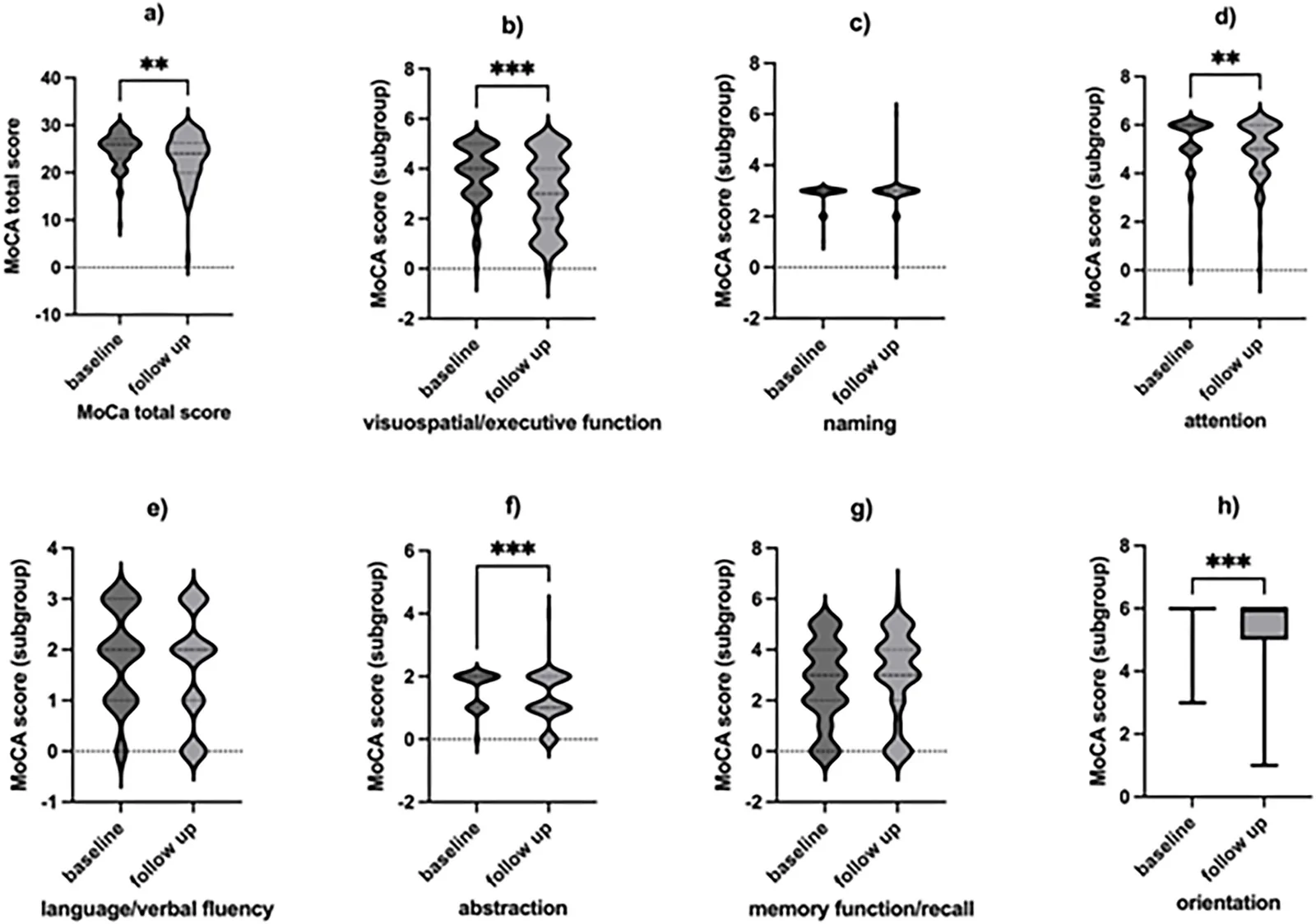
Changes in MoCA total and domain-specific subscores from baseline to follow-up. Violin plots illustrate the distribution of scores at baseline and follow-up for (a) total MoCA score, (b) visuospatial/executive function, (c) naming, (d) attention, (e) language/verbal fluency, (f) abstraction, (g) memory/recall, and (h) orientation. Overall, a significant decline in total MoCA score is observed over time (a). Domain-specific analyses reveal significant changes in visuospatial/executive function (b), attention (d), abstraction (f), and orientation (h), whereas naming (c), language/verbal fluency (e), and memory/recall (g) remain relatively stable without statistically significant differences. The shape of each violin plot reflects the score distribution, with central markers indicating median values. Statistical significance is denoted as **p < 0.01 and ***p < 0.001.

**Table 2:** MoCA total score and subcategories at baseline and follow up.

	Baseline	Follow up	Maximum	*P*-value^a^
MoCA total score, mean ± SD	24.8 ± 3.7	23.0 ± 4.9	30	**<0.001**
Visuospatial/executive function, mean ± SD	3.8 ± 1.2	3.1 ± 1.5	5	**<0.001**
Naming, mean ± SD	2.9 ± 0.4	2.9 ± 0.5	3	0.59
Attention, mean ± SD	5.4 ± 1.0	5.0 ± 1.2	6	**0.008**
Language/verbal fluency, mean ± SD	1.9 ± 0.9	1.6 ± 1.1	3	0.16
Abstraction, mean ± SD	1.6 ± 0.6	1.3 ± 0.8	2	**<0.001**
Memory function/recall, mean ± SD	2.7 ± 1.6	3.0 ± 1.7	5	0.14
Orientation, mean ± SD	5.9 ± 0.4	5.6 ± 0.9	6	**<0.001**

a Calculation with Mann–Whitney test.

### Risk factors for MoCA decline

The mean MoCA difference between baseline and follow-up was −1.8 ± 3.2. Among the 90 patients, 53 (59%) were classified as cognitively stable (sCF), while 37 (41%) exhibited cognitive decline (dCF) based on a change in MoCA score >2 points over time. There was a notable contrast in MoCA difference between sexes, with males having a median (range) of −3.0 (0.0 to −5.0) and females having a median (range) of 0.0 (1.0 to −1.75) points MoCA difference, which was statistically significant (*P* = 0.002). This association was confirmed when comparing the sCF and dCF groups (Table 3, *P* < 0.001), with 92% of patients in the dCF group being male (Figs 3 and 4). After adjustment for age, educational level, diabetes, and blood pressure readings, this association remained significant (*P* = 0.006, regression coefficient −2.5 for the MoCA difference, and *P* = 0.007 with a regression coefficient of 2.65 and odds ratio of 14.2 for the between-group comparison).

**Figure 3:**
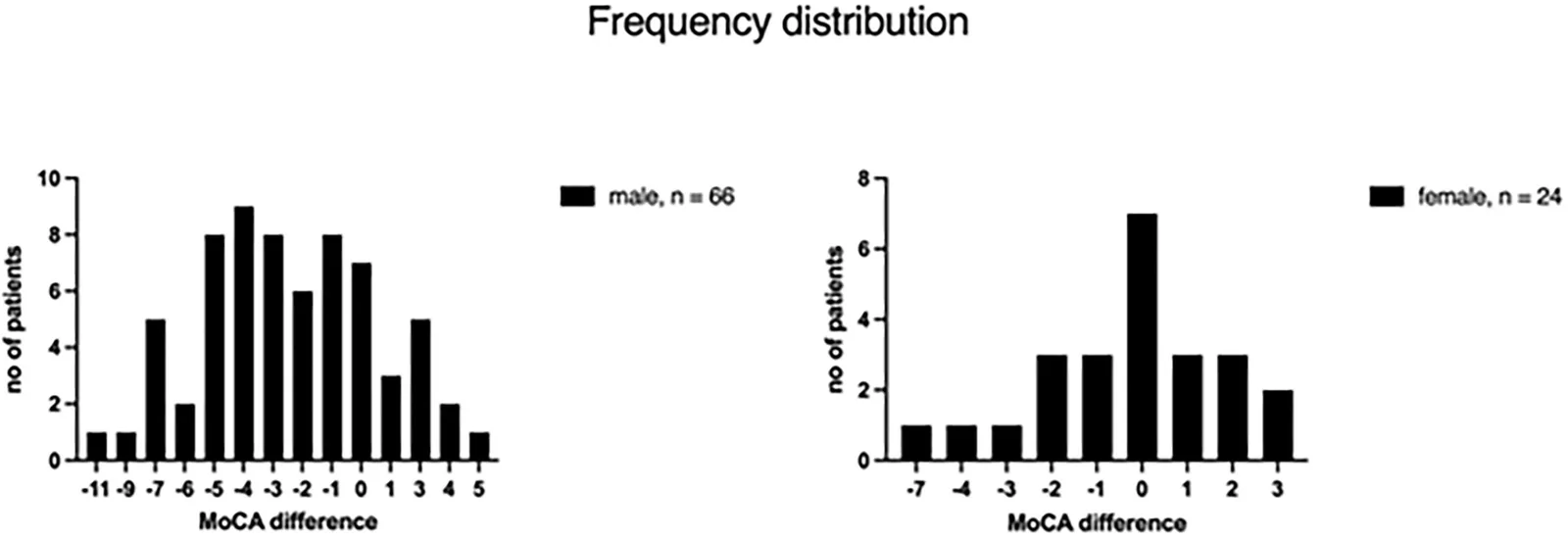
Frequency distribution of MoCA difference stratified by sex. Histograms display the distribution of differences between follow-up and baseline MoCA total scores for male (n = 66) and female (n = 24) patients. The x-axis represents the change in MoCA score, with negative values indicating cognitive decline and positive values indicating improvement, while the y-axis denotes the number of patients. In males, score changes span a wider range, with a notable concentration of declines between −5 and 0 points. In females, the distribution is narrower, with most changes clustering between −2 and +2 points.

**Figure 4:**
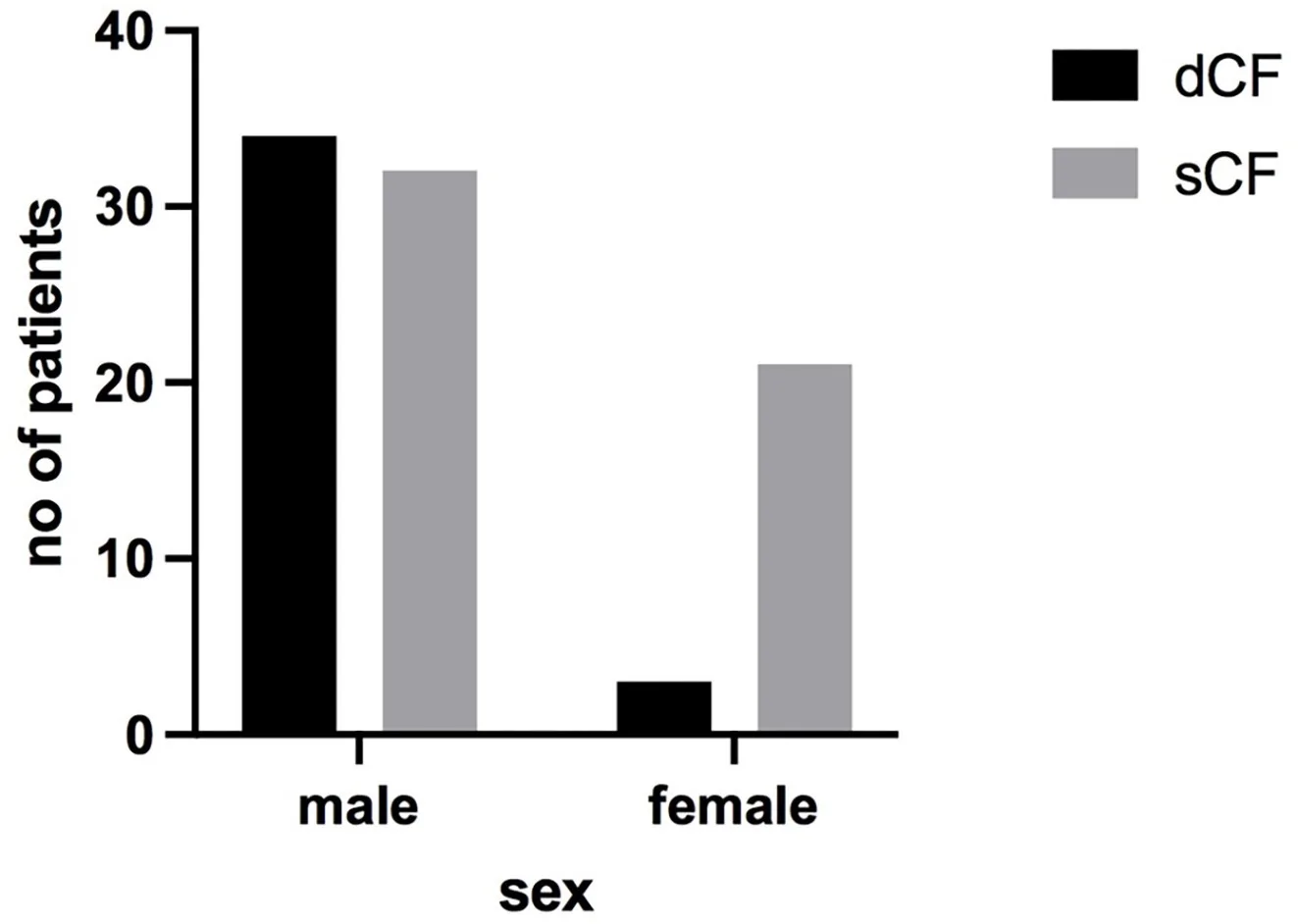
Sex-specific distribution of cognitive trajectories. Bar charts illustrate the number of patients classified as declining cognitive function (dCF) or stable cognitive function (sCF), stratified by sex. In male patients, the proportions of dCF and sCF are relatively balanced. In contrast, female patients predominantly exhibit stable cognitive function, with only a small proportion classified as dCF. The y-axis indicates the number of patients, and the x-axis denotes sex.

**Table 3:** Factors associated with cognitive decline.

	dCF	sCF	*P*-value^a,b,c^
**Traditional risk factors**			
Age (years), mean ± SD	65.7 ± 14.3	60.5 ± 14.9	0.12^b^
Sex m/f, *n* (%)	34 (92)/3 (8)	32 (60)/21 (40)	**<0.001^c^**
Educational level <12 years, *n* (%)	23 (62)	27 (51)	0.29^c^
Arterial hypertension, *n* (%)	34 (92)	47 (89)	0.62^c^
Diabetes mellitus, *n* (%)	12 (32)	14 (26)	0.53^c^
Hypercholesterolemia, *n* (%)	19 (51)	28 (53)	0.89^c^
Current smokers, *n* (%)	5 (14)	11 (21)	0.37^c^
**Dialysis associated risk factors**			
Dialysis vintage (months), mean ± SD	47 ± 46	79 ± 93	0.09^b^
Time on dialysis (h), mean ± SD	4.4 ± 0.8	4.7 ± 1.1	0.40^b^
Ultrafiltration volume (l), mean ± SD	1.5 ± 0.8	2.0 ± 1.1	0.06^a^
Kt/V, mean ± SD	1.6 ± 0.5	1.6 ± 0.4	0.24^b^
**Risk factors for vascular calcification**			
Calcium (mmol/l), mean ± SD	2.2 ± 0.3	2.3 ± 0.2	0.27^b^
Phosphate (mmol/l), mean ± SD	5.2 ± 1.5	5.4 ± 1.3	0.46^a^
PTH (pg/ml), mean ± SD	332 ± 323	239 ± 190	0.59^b^
T_50_ (min), mean ± SD	228 ± 71	255 ± 79	0.11^a^
**Hemodynamics**			
HR (bpm), mean ± SD	72 ± 10	73 ± 11	0.70^b^
pSBP (mmHg), mean ± SD	125 ± 11	120 ± 15	0.11^a^
pMAP (mmHg), mean ± SD	99 ± 10	94 ± 12	**0.04^a^**
pDBP (mmHg), mean ± SD	76 ± 11	73 ± 10	0.24^a^
PWV (m/s), mean ± SD	9.6 ± 2.0	8.7 ± 2.2	0.09^b^

Calculation with ^a^unpaired *t*-test, ^b^Mann–Whitney-*U*-test, ^c^*χ*^2^-test.

All other traditional risk factors, dialysis-associated risk factors and parameters of vascular calcification did not show significant differences in both group comparisons. However, a trend toward higher ultrafiltration volume was observed in the comparison between the sCF and dCF groups (*P* = 0.06), with higher values in the sCF group (2.0 ± 1.1 vs. 1.5 ± 0.8 l in the dCF group). Concerning hemodynamics, the mean arterial pressure was significantly different in sCF and dCF patients (*P* = 0.04, Table 3), with patients experiencing cognitive decline showing slightly higher values than those in the sCF group. However, statistical significance was lost when adjusting for covariates (*P* = 0.273).

## DISCUSSION

In the present study, our focus centered on elucidating the trajectory of cognitive function and its determinants in hemodialysis patients over time. We incorporated PWV as a surrogate for arterial stiffness and T50 as a biomarker for calcification propensity, aiming to address the high cardiovascular burden in the end-stage renal disease (ESRD) patient population and its potential role in the progression of cognitive impairment. Our findings revealed a significant decline in cognitive function, as measured by the MoCA, after an ∼2-year interval. Identified risk factors for progressive cognitive function decline included male sex and elevated mean arterial blood pressure, with male sex retaining significance in adjusted analyses.

Over the study duration, cognitive function deteriorated significantly, with an average decrease of 1.8 points in the MoCA scores. Previous studies assessing MoCA scores over time in the general population have produced contradictory results [22, 23], demonstrating varying degrees of (age-dependent) MoCA score decline and even increases. Notably, a study on changes in MoCA scores over time in patients with cerebral small vessel disease and mild cognitive impairment (MCI) reported a smaller decrease of 1.3 points after 2 years, despite the older age of the cohort (mean age 74.4 years) and preexisting MCI diagnosis [24]. Studies focusing on cognitive function decline in hemodialysis patients using the MoCA test are scarce. Two studies investigating cerebral blood flow and cognitive decline in hemodialysis patients assessed cognitive performance using the MoCA at baseline and after 1 year [5, 8]. Their findings were inconsistent: one study reported a decline of 0.6 points, comparable to that observed in the present study, whereas the other demonstrated an improvement of 2 points. The younger age of the latter cohort (median age 59 years) may partly account for this difference. Studies utilizing the MMSE and neurocognitive batteries have likewise yielded mixed results. Some studies found declines in cognitive function [2–4], others reported stable cognition or improvement [6, 7], possibly explained by shorter follow-up intervals.

A notable strength of our study lies in the comprehensive integration of a wide range of risk factors, including markers of arterial stiffness and vascular calcification, not previously studied in this context. The sole independent risk factor identified was sex, with male sex associated with a greater decline in cognitive function over time. Besides age, sex represents a pivotal risk factor for cognitive impairment and dementia in the general population, where female sex poses a two-fold increased risk for Alzheimer’s disease development, while males face a higher risk of vascular dementia [25, 26]. The consistency of male sex as a risk factor with the (suspected) vascular origin of cognitive impairment in hemodialysis patients aligns with current research and understanding of the disease. To date, only older age and frailty have been identified as risk factors for cognitive function deterioration in hemodialysis patients [2, 4]. Further studies, with larger patient samples and extended follow-up periods, are imperative to evaluate these and additional risk factors.

A noteworthy observation was the trend toward higher ultrafiltration volumes in the group with preserved cognitive function. Studies investigating cerebral blood flow during hemodialysis sessions have demonstrated a significant reduction in cerebral perfusion, which was associated with cumulative ultrafiltration volume [5, 8]. However, as discussed above, the impact on cognitive performance remains unclear.

Our study has several limitations. First, there are two potential sources of selection bias. Patients with more pronounced cognitive impairment or established dementia were generally not included, which may have led to an underestimation of both the prevalence and progression of cognitive impairment. In addition, fewer than half of the participants underwent follow-up cognitive testing. This was partly attributable to the high mortality rate in this population, but also to withdrawal of consent. It is conceivable that patients experiencing a more pronounced cognitive decline were less likely to participate in repeated assessments. The sensitivity analyses underscore the potential impact of mortality-related missing data on estimates of cognitive decline and should be considered when interpreting the magnitude of the observed changes.

The second limitation relates to the choice of the cognitive assessment instrument, as well as its application and interpretation within the study. While a neuropsychological test battery could offer a more comprehensive understanding of cognitive status, the MoCA test, given its appropriateness for ESRD patients and low time requirement, was a practical choice for our trial. The restriction of cognitive testing to native speakers may have compromised the external validity of the study and limits the applicability of the findings to the wider hemodialysis population. The chosen cutoff of 2 points for discriminating cognitively stable and declining patients may be considered arbitrary but supporting data in the general population (and in hemodialysis patients) are lacking. However, in patients with a previous transient ischemic attack (TIA) or stroke, one study found that a decline of ≥2 points on the MoCA was associated with a significant deterioration in a neuropsychological test battery. This threshold was subsequently applied and supported by findings from a large observational study in a similar patient population [27, 28]. Our primary approach, utilizing MoCA difference, also aims to mitigate this limitation.

The absence of neuroimaging techniques to assess structural and functional brain alterations constitutes an additional limitation, as it precludes a more comprehensive evaluation of the mechanisms underlying cognitive changes.

In conclusion, our study demonstrates a significant decline in cognitive function among hemodialysis patients, consistent with previous findings. Although we explored a broad spectrum of potential risk factors, including parameters of arterial stiffness and vascular calcification for the first time, we could not conclusively establish their involvement, likely due to the small cohort size and high loss to follow-up. Nonetheless, this study is the first to associate male sex with accelerated cognitive function decline in hemodialysis patients. This finding is consistent with the well-established association between male sex and vascular dementia in the general population. However, it remains unclear whether the observed association in hemodialysis patients is attributable to vascular dementia. Further research is needed to validate these findings and to clarify the mechanisms involved. A deeper understanding of the driving factors of cognitive impairment in hemodialysis patients is essential for developing preventive and therapeutic strategies.

## Supplementary Material

sfag340_Supplemental_File

## Data Availability

The data underlying this article will be shared on reasonable request to the corresponding author.
