# Supplementary material for: Longitudinal assessment of cognitive decline in hemodialysis patients: unveiling sex disparities
Source: Clin Kidney J. 2026 Oct 7;19(11):sfag340. doi: 10.1093/ckj/sfag340 (PMC13724265; doi:10.1093/ckj/sfag340)
Supplement: Supplementary file 1 [file sfag340_supplemental_file.docx]

Supplementary Table 1: Demographic and clinical characteristics and factors associated with cognitive decline divided in a group with and without follow up.

| **Traditional risk factors** | Patients with follow up | Patients without follow up | p-value^a,b^ |
| --- | --- | --- | --- |
| Age (years), mean ± S.D. | 63.3 ± 14.8 | 66.5 ± 15.1 | 0.107a |
| Sex m/f, n (%) | 64 (73) / 24 (27) | 111 (71) / 46 (29) | 0.736c |
| Educational level < 12 years, n (%) | 49 (55) | 103 (66) | 0.125c |
| Arterial hypertension, n (%) | 83 (94) | 149 (95) | 0.844^b^ |
| Diabetes mellitus, n (%) | 28 (32) | 66 (42) | 0.115^b^ |
| Hypercholesterolemia, n (%) | 51 (58) | 111 (71) | 0.043^b^ |
| Current smokers, n (%) | 18 (20) | 33 (21) | 0.917^b^ |
| **Dialysis associated risk factors** |  |  |  |
| Dialysis vintage mean ± S.D. | 67 ± 79 | 58 ± 51 | 0.353^a^ |
| Time on dialysis (hours), mean ± S.D. | 4.6 ± 1.0 | 4.4 ± 0.6 | 0.086^a^ |
| Ultrafiltration volume (liters), mean ± S.D. | 1.8 ± 1.0 | 1.6 ± 1.3 | 0.312^a^ |
| Kt/V, mean ± S.D. | 1.65± 0.4 | 1.5 ± 0.3 | 0.101^a^ |
| **Risk factors for vascular calcification** |  |  |  |
| Calcium (mmol/l), mean ± S.D. | 2.2 ± 0.2 | 2.3 ± 0.2 | 0.056^a^ |
| Phosphate (mmol/l), mean ± S.D. | 1.7 ± 0.5 | 1.6 ± 0.5 | 0.261^a^ |
| PTH (pg/ml), mean ± S.D. | 299 ± 237 | 286 ± 285 | 0.727^a^ |
| T_50_ (min), mean ± S.D. | 244 ± 77 | 249 ± 74 | 0.591^a^ |
| **Hemodynamics** |  |  |  |
| HR (bpm), mean ± S.D. | 73 ± 11 | 72 ± 11 | 0.480^a^ |
| pSBP (mmHg), mean ± S.D. | 122 ± 14 | 124 ± 17 | 0.437^a^ |
| pMAP (mmHg), mean ± S.D. | 96 ± 11 | 96 ± 14 | 0.848^a^ |
| pDBP (mmHg), mean ± S.D. | 74 ± 11 | 73 ± 13 | 0.459^a^ |
| PWV (m/s), mean ± S.D. | 9.1 ± 2.2 | 9.7 ± 2.2 | 0.074^a^ |
| MoCA total score | 24.3 ± 3.8 | 23.4 ± 4.1 | 0.056^a^ |

Abbreviations: bpm, beats per minute; HR, heart rate; m/f, male/female; pDBP, 24h peripheral diastolic blood pressure; pMAP, 24h peripheral mean arterial pressure; pSBP, 24h peripheral systolic blood pressure; PWV, pulse wave velocity; T_50_, T_50_-test

^a,b,c^ calculation with ^a)^ unpaired t-test, ^b))^ χ^2^-test

Supplementary Table 2: Sensitivity analyses of longitudinal changes in cognitive function assessed by the Montreal Cognitive Assessment (MoCA).

| **Analysis** | **N** | **Mean change in MoCA** | **SD** | **Minimum** | **Maximum** |
| --- | --- | --- | --- | --- | --- |
| Complete-case analysis | 90 | -1.82 | 3.14 | -11 | 5 |
| Sensitivity analysis: deceased participants assigned MoCA = 0 | 127 | -7.93 | 10.17 | -30 | 5 |
| Sensitivity analysis: deceased participants assigned 50% of baseline MoCA | 127 | -4.61 | 5.22 | -15 | 5 |
| Sensitivity analysis: last observation carried forward (LOCF) | 127 | -1.29 | 2.76 | -11 | 5 |

Abbreviations: LOCF, last observation carried forward.
